# Proposal of a new chemical marker for the quality control of the herb *Scleromitrion diffusum*

**DOI:** 10.3389/fchem.2025.1600769

**Published:** 2025-04-29

**Authors:** Tao Zheng, Chu-Chu Zhong, Grace Gar-Lee Yue, Dong-Min Jiang, Man-Ho Tong, Hiu-Yan Wong, Kwan-Ho Wong, Meng-Hua Wu, David Tai-Wai Lau, Hui Cao, Pang-Chui Shaw, Clara Bik-San Lau

**Affiliations:** ^1^ Institute of Chinese Medicine, The Chinese University of Hong Kong, Shatin, New Territories, Hong Kong SAR, China; ^2^ State Key Laboratory of Research on Bioactivities and Clinical Applications of Medicinal Plants, The Chinese University of Hong Kong, Shatin, New Territories, Hong Kong SAR, China; ^3^ College of Pharmacy, Jinan University, Guangzhou, China; ^4^ Li Dak Sum Yip Yio Chin R & D Centre for Chinese Medicine, The Chinese University of Hong Kong, Shatin, New Territories, Hong Kong SAR, China; ^5^ Department of Pharmacology and Pharmacy, LKS Faculty of Medicine, The University of Hong Kong, Pokfulam, Hong Kong SAR, China; ^6^ Shiu-Ying Hu Herbarium, School of Life Sciences, The Chinese University of Hong Kong, Shatin, New Territories, Hong Kong SAR, China; ^7^ School of Life Sciences, The Chinese University of Hong Kong, Shatin, New Territories, Hong Kong SAR, China; ^8^ School of Chinese Medicine, LKS Faculty of Medicine, The University of Hong Kong, Pokfulam, Hong Kong SAR, China

**Keywords:** *Hedyotis diffusa*, *Scleromitrion diffusum*, thin layer chromatography, highperformance liquid chromatography, pharmacopoeia chemical marker, quality control

## Abstract

Baihuasheshecao (*Scleromitrion diffusum* (Willd.) R.J. Wang), a traditional Chinese medicinal herb, is extensively used as an ingredient in herbal teas and is a well-known folk medicine in China. However, two common adulterants, *Hedyotis corymbosa* (L.) Lam*.* and *Hedyotis tenelliflora* (BL.) Kuntze, belonging to the same family, are frequently mistaken as *S. diffusum* in folk medicine. This confusion has led to a growing concern about their identification and quality evaluation. While previous studies have demonstrated over 15 various compounds as potential chemical markers for authentication and quality control for *S. diffusum*, considering the selection criteria for chemical markers in the Chinese Pharmacopoeia, most of these compounds do not meet the pharmacopoeial requirements. Hence, this study aims to identify an appropriate chemical marker for *S. diffusum* based on thin layer chromatography (TLC) identification and high-performance liquid chromatography (HPLC) quality control, in order to differentiate from any counterfeits such as *H. corymbosa* and *H. tenelliflora*. Our findings suggested for the first time that the compound (*E*)-6-*O*-(*p*-coumaroyl) scandoside methyl ester which is specific to *S. diffusum*, could serve as the chemical marker. Here, attempt was made to establish standardized protocols for TLC identification, HPLC analysis, and quantitative methodologies, adhering to Chinese pharmacopoeial standards, along with laboratory scale preparation procedures. In conclusion, our findings have provided valuable insights to the identification and quality control processes for *S. diffusum*, and anticipated with potential applications by the industry and regulatory authority.

## Introduction

Baihuasheshecao, whose former Latin name is *Hedyotis diffusa* Willd., is an annual slender plant which belongs to the Rubiaceae family. Since 1798, when Willdenow proposed first named the species *H. diffusa* Willd., the scientific nomenclature of Baihuasheshecao has undergone several changes. In 1814, Roxburgh reclassified the species under the genus *Oldenlandia*, resulting in the name *Oldenlandia diffusa* (Willd.) Roxb. Most recently, in 2014, Wang reassessed the taxonomy based on morphological and molecular data, renaming the species *Scleromitrion diffusum* (Willd.) R. J. Wang. This final naming was confirmed with the proper selection of a lectotype in 2021 (Siu et al., 2021). Therefore, this paper will use *S. diffusum* as the Latin name for Baihuasheshecao.

Modern pharmacological studies have corroborated its diverse biological activities, such as neuroprotection, antioxidant, anti-cancer, anti-inflammatory, antibacterial, and immunomodulating activities (Chen R. et al., 2016; Chen Y. et al., 2016; Gao et al., 2016; Kuo et al., 2015; Park and Whang, 2020; Lin et al., 2011; Rui and Zhao, 2002). As a revered traditional Chinese medicinal herb, it is extensively used for the treatment of various types of diseases, including colon cancer, liver cancer, bronchitis, arthritis, rheumatism, appendicitis, sore throat, urethral infection, contusions, ulcerations and malignancy extension (Chen R. et al., 2016; Niu and Meng, 2013; Yu et al., 2002). According to the data from the National Health Insurance Research Database of Taiwan, this single Chinese herbal medicine has been proven as the most commonly prescribed used for colon cancer and breast cancer patients (Chao et al., 2014; Yeh et al., 2014). Furthermore, more than 170 compounds have been isolated from Baihuasheshecao, including iridoid glycosides, triterpenoids, flavonoids, anthraquinones, phenolic acids and their derivatives, sphingolipids, sterols, alkaloids, volatile oils and polysaccharides (Chen R. et al., 2016; Kim et al., 2001; Li et al., 2010; Lu and He, 1996; Ren et al., 2005). Among these, flavonoids, anthraquinones, triterpenes and iridoids, as the main constituents of Baihuasheshecao are also considered as the major bioactive constituents (Kim et al., 2001; Li et al., 2010; He et al., 2018).

In traditional folk medicine, Baihuasheshecao is commonly prepared as “liangcha,” a herbal tea or decoction used for its heat-clearing, detoxifying and dampness-eliminating properties. These remedies are popular in the herb’s native regions, particularly in southern China, including Guangdong, Hong Kong, Macau, and tropical Asia (Li et al., 2010). Beyond its folk usage, Baihuasheshecao also features prominently in Chinese patent medicines, with 17 formulations listed in the Chinese Pharmacopoeia, including Nankang pian, Huahong pian, Yiganning keli, and Shuanghuqinggan keli (National Pharmacopoeia Committee, 2020). The Baihuasheshecao injection, utilized for clearing heat, detoxification, dampness elimination, and as an adjuvant treatment for cancer, has been on the market for many years (Xu et al., 2008).

In recent years, researches for authentication and pharmacognosy on Baihuasheshecao had become more important, especially in differentiating its genuine species and related adulterated species such as *Hedyotis corymbosa* and *Hedyotis tenelliflora*. *H. corymbosa*, a species belonging to the same family as Baihuasheshecao is also frequently misidentified and sold as Baihuasheshecao in the medicinal and food markets in Hong Kong and Guangdong province (Liang et al., 2007). Although they share similarities in morphology, they do exhibit differences in terms of their chemical constituents and medicinal properties, especially the anti-tumor activities of *S. diffusum* are more potent than those of *H. corymbosa* (Lau et al., 2012). To make it more complicated, another species, *H*. *tenelliflora*, resembling *S. diffusum* in appearance, has also been misidentified and used as Baihuasheshecao in Yunnan and Guangdong provinces, but the functions and efficacies of these two herbs are not quite the same (Lin et al., 2004).

To ensure the effective and correct use of Baihuasheshecao, it is imperative to identify a specific chemical marker and establish reliable methods for the authentication and quality control of Baihuasheshecao. Previous studies on the identification and quality control of *S. diffusum* and similar species have focused on the chemical profiling using modern analytical techniques such as HPLC, HPLC-MS, GC-MS, UPLC-QTOFMS, and even other techniques such as nuclear magnetic resonance-mass spectrometer (NMR-MS), DNA sequencing methods and X-ray diffraction methods (Chen R. et al., 2016; Li et al., 2010; Liang et al., 2007; Wang et al., 2018; Liu et al., 2003; Wang et al., 2003; Li et al., 2013). However, these methods necessitate specialized equipment and are yet to be widely adopted by the Chinese Pharmacopoeia (Shen et al., 2021). Up to now, over 15 various compounds have been identified as potential chemical markers for authenticating and controlling the quality of *S. diffusum* and distinguishing it from substitutes (Wang et al., 2018). These chemical markers encompass various iridoids (deacetyl asperulosidic acid methyl ester, scandoside methyl ester, asperuloside, asperuloside acid, geniposidic acid, (*E*)-6-*O*-(*p*-coumaroyl) scandoside methyl ester, and (*E)*-6-*O*-(*p*-coumaroyl) scandoside methyl ester-10-*O*-methyl ether), triterpenes (oleanolic acid, ursolic acid), phenolic acids (*p*-coumaric acid, ferulic acid), flavonoids (rutin, quercetin, kaempferol), hedyotiscone A and aesculetin (Park and Whang, 2020; Liang et al., 2007; Lau et al., 2012; Wang et al., 2018; Liang et al., 2006). However, many of these compounds were also commonly found in other herbs, including *H. corymbosa* and *H. tenelliflora* (Wang et al., 2018; Liang et al., 2006; Waghdhare, 2021), which limits their effectiveness in terms of species identification and quality control. In addition, some of these compounds are present in low concentrations in *S. diffusum* and hence are not representative of the main components, making them unsuitable as chemical markers.

The aim of this study is to identify an appropriate chemical marker from *S. diffusum* which can be used to differentiate from its common adulterants *H. corymbosa* and *H*. *tenelliflora.* To achieve this, we endeavor to develop TLC and HPLC methods under optimized conditions tailored for the identification and quality control of *S. diffusum*. Furthermore, our research team has endeavored to establish a process for the large-scale isolation and purification of this chemical marker from *S. diffusum* in the laboratory setting, plus some preliminary stability studies on this compound.

## Materials and methods

### Chemical reagents

Analytical grade methanol, ethanol, ethyl acetate, petroleum ether, n-butanol (Labscan, Bangkok, Thailand) were used for sample extraction, partition, TLC analysis, and open column chromatography. Chromatography-grade acetic acid and acetonitrile were purchased from Thermo Fisher Scientific (Fair Lawn, NJ, United States). HPLC-grade methanol was obtained from Dulsan Reagents (Dulsan Pure Chemicals Co. Ltd., Korea)., while ultrapure water was prepared with a Milli-Q system (Millipore, France). Chemical reference standards of asperuloside (Batch number: 20071726) was purchased from Shanghai Tauto Biotech Co. Ltd. (*E*)-6-*O*-(*p*-coumaroyl) scandoside methyl ester (Batch number: RFS-D10802303010) was purchased from Chengdu Herbpurify Co. Ltd., and the standard herb (*S. diffusum*, Batch number: 121183-201605; ID:CFF3-Y7EL) was supplied by the National Institute for the Control of Pharmaceutical and Biological Products (Beijing, China). A self-made reference standard of (*E*)-6-*O*-(*p*-coumaroyl) scandoside methyl ester was isolated and purified from the whole dried herb of *S. diffusum* in our laboratory. The structure was elucidated on the basis of single-crystal x-ray diffraction, NMR spectral data and the purity of the compound measured by HPLC-DAD was shown to be greater than 98%.

### Plant materials

The whole dried herb used for extracting and isolating the compound was purchased from Zisun Hong Kong Limited (China) in 2023. A total of thirty batches of *S. diffusum* herbal materials and sixteen batches of *S. diffusum* decoction pieces were purchased from different herbal markets or traditional Chinese medicine companies across China. Additionally, two batches of *H. corymbosa* and two batches *H. tenelliflora* herbal materials were collected from different origins in China. Four batches of fresh *S. diffusum* and four batches of fresh *H. corymbosa* herbs were also collected from various habitats in Hong Kong (Supplementary Table S1). All plant materials were subjected to morphological authentication by Dr. David Tai-Wai Lau of Shiu-Ying Hu herbarium in The Chinese University of Hong Kong, and the corresponding voucher specimens were deposited at the museum of Institute of Chinese Medicine, The Chinese University of Hong Kong. Furthermore, all authenticated fresh herbs were subjected to standardized specimen processing methods and were deposited in the Shiu-Ying Hu herbarium as voucher specimens with inventory numbers. Fresh herbal materials for TLC and HPLC chemical analysis were reserved and dried immediately in an oven at 55°C for 24 h for each specimen.

### Equipment

Column chromatography was performed using D101 (10-60 mesh, Tianjin Haiguanghuagong, China), silica gel (40-63 μm, Merck, Germany), and Sephadex LH-20 (GE Healthcare Bio-Sciences AB, Sweden). Compounds were prepared using a preparative HPLC (LC-52, Separation, Beijing, China) equipped with an ODSA preparative chromatography column (YMC-pack, 5 μm, 12 nm, 20 × 250 mm, Japan), Agilent 1290 Infinity UHPLC system coupled to an Agilent 6530 Accurate-Mass Quadrupole Time-of-Flight Mass Spectrometer with Dual Electrospray Ionization Source (Agilent, CA, United States). Bruker Avance-500 MHz NMR instrument (Bruker Corporation, Germany) was utilized to provide all the NMR spectra, including 1D-NMR and 2D-NMR spectra, and the chemical shifts were referenced to tetramethylsilane (TMS). X-ray crystallographic data were collected on an Oxford Dual, Cu at zero, Atlas S2 diffractometer (Rigaku Corporation, Japan). TLC analysis was carried out using Silica gel 60 F_254_ (TLC) plates (Merck, Germany). MS-grade methanol (MeOH) and acetonitrile (ACN) were obtained from RCI Labscan, Ltd. (Bangkok, Thailand).

### Preparation of sample and standard solutions for TLC and HPLC

All herbal batches were grounded to powder and passed through a 24-mesh screen separately. 2.0 g of the sample powder was precisely weighed and extracted with 20 mL of 70% ethanol for 80 min under reflux conditions. Following centrifugation at 4,000 rpm for 5 min, the supernatant was then filtered through a 0.45 μm membrane before TLC and HPLC analysis.

Asperuloside and (*E*)-6-*O*-(*p*-coumaroyl) scandoside methyl ester were weighed and dissolved in methanol to produce standard stock solutions at concentrations of 300 μg/mL and 400 μg/mL, respectively. For quantitative analysis, a mixed reference solution (in methanol) containing 4 compounds: deacetyl asperulosidic acid methyl ester, scandoside methyl ester, asperuloside and (*E*)-6-*O*-(*p*-coumaroyl) scandoside methyl ester, at concentrations of 14.8, 53.0, 137.5, 196.4 μg/mL was prepared and stored at 4°C prior to use.

### TLC chromatographic conditions

For each herbal sample and standard solution, bandwise application was performed using an ATS4 auto-sampler (CAMAG, Muttenz, Switzerland) on a commercial 20 cm × 10 cm pre-coated TLC silica gel 60 F_254_ plate (Merck, Germany). The application conditions were as follows: carrier gas, compressed air; syringe delivery speed, 150 nL/S; application volume, 5 μL; band length, 7 mm; space between two bands, 5 mm; distance from bottom, 10 mm. The sample-loaded plate was dried under hot air and placed into a desiccator with color changing silica gel. Twenty milliliters of mobile phase consisting of ethyl acetate: methanol: water (8: 1.5: 0.7, v/v/v) was added into a twin-trough chamber and saturated for 15 min. The plate in the chamber was then developed upward over a path of 8 cm at room temperature (22°C). Developed plates were dried for 3 min with hairdryer. The excitation wavelength was set at 254 nm in reflection mode, and the exposure time was 0.5 s. The plate was then heated on a TLC plate heater (CAMAG, Muttenz, Switzerland) at approximately 105°C after spraying with 10% sulfuric acid in ethanol until the color of the spots appeared distinctly. High-definition images of the TLC plate were captured using a Visualizer 3 (CAMAG, Muttenz, Switzerland) linked with WinCATS software under visible light.

### HPLC chromatographic conditions for quantification

Chromatographic condition for components quantification was performed on an Agilent 1260 Infinity series HPLC system (Santa Clara, California, United States) coupled with Agilent Eclipse Plus C_18_ (5 μm, 4.6 × 250 mm). The UV spectra (DAD) were determined to be 240 nm. The solvent system comprised of a 0.2% acetic acid aqueous solution (A) and acetonitrile (B) with the following gradient program: 5% B for 0–10 min, 5%–10% B for 10–20 min, 10%–15% B for 20–45 min, 15%–20% B for 45–60 min, 20% B for 60–80 min, and the post-run (5 min) at a flow rate of 0.8 mL/min. The injection volume was 5 μL, and the column temperature was maintained at 30 °C.

### TLC guided extraction, isolation and preparation of chemical marker compound

(*E*)-6-*O*-(*p*-coumaroyl) scandoside methyl ester, an iridoid glycoside compound, was extracted based on its chemical properties and the extraction method for medicinal materials, as outlined below (also see Supplementary Figure S1).

The *S. diffusum* decoction pieces (2.4 kg) were extracted using 70% ethanol under reflux three times (24 L × 1 h × 3 times), filtered, and the filtrate was combined and concentrated to dryness to give a condensed extract (160 g). The dried residue was then resuspended in 3-4 L of distilled water and successively partitioned with equal volume of petroleum ether (five times) and butanol (six times), respectively. TLC analysis of all three fractions revealed the presence of the marker compound in the butanol fraction, which was concentrated to dryness, resuspended in water, and passed through a D101 resin column (inner diameter 15 cm, column height 50 cm). The column was then eluted sequentially with water (7 L), 20% ethanol (7 L), and 60% ethanol (7 L), respectively to afford three fractions (Fr. B1- Fr. B3). As indicated by TLC tracking, Fr. B2 was further separated on a Sephadex LH-20 column with a methanol eluent to give four fractions (Fr. B21- Fr. B23). Similarly, the eluates were monitored by TLC and Fr. B22 was subjected to silica gel column chromatography (200-300 mesh, inner diameter 3 cm, column height 40 cm) and eluted with a gradient of 900 mL of ethyl acetate, followed by ethyl acetate-methanol (12:1, v/v, 900 mL) and ethyl acetate-methanol (8:1, v/v, 900 mL) to give three subfractions (Fr. B221- Fr. B223).

The targeted subfraction was then redissolved in methanol, filtered, and subjected to a preparative liquid chromatography system (PHPLC, RP-18 column, 20 mm × 250 mm, 5 μm particle size, 10 mL/min flow rate). The compound was eluted with 23% v/v acetonitrile in water, and the appropriate fraction (Rt = 21 min) was collected and evaporated to obtain the chemical marker. However, HPLC analysis revealed that the chemical purity was still insufficient (below 98%). Fortunately, we observed that the compound exhibited a tendency to crystallize under specific conditions. To enhance the purity, the compound was redissolved in an appropriate amount of methanol to make a supersaturated solution (with appropriate heating or ultrasonic if necessary). Five volumes of distilled water were then added, and the mixture was kept in an ice bath for 20 min. Subsequently, centrifugation at 4,000 rpm for 10 min was performed to separate the precipitate from the supernatant. This process was repeated for the obtained precipitate until no impurities were detected in the mother liquor. After ten cycles of recrystallization, 4.8 g of the final product was obtained, corresponding to a yield of 0.2%. Encouragingly, subsequent HPLC purity analysis revealed that the purity of the marker now exceeded 98%, surpassing even the purity of commercially available chemical markers.

### Spectrum data and chemical structure of (*E*)*-*6*-O-*(*p*-coumaroyl) scandoside methyl ester

The chemical compound was identified as (*E*)-6-*O*-(*p*-coumaroyl) scandoside methyl ester by the methods of HR-ESIMS, ^1^H-NMR, ^13^C-NMR (Supplementary Figure S2). The spectrum data and chemical structure of the compound were as follows: C_26_H_30_O_13;_ HR-ESIMS *m/z* [M - H]^-^ 549.1625 (calcd. for C_26_H_29_O_13_, 549.1608). ^1^H NMR (500 MHz, Methanol-*d*_4_); *δ* 7.61 (1H, d, *J* = 15.9 Hz, H-7′), 7.49 (1H, d, *J* = 1.1 Hz, H-3), 7.45 (2H, d, *J* = 8.7 Hz, H-2′, H-6′), 6.79 (2H, d, *J* = 8.7 Hz, H-3′, H-5′), 6.32 (1H, d, *J* = 15.9 Hz, H-8′), 5.83 (1H, p, *J* = 1.8 Hz,H-7), 5.66 (1H, dt, *J* = 4.8, 1.8 Hz, H-6), 5.29 (1H, d, *J* = 7.0 Hz, H-1), 4.68 (1H, d, *J* = 7.8 Hz, H-1″), 4.37 (1H, dt, *J* = 15.6, 1.8 Hz, H-10), 4.20 (1H, dt, *J* = 15.9, 2.0 Hz, H-10), 3.87 (2H, dt, *J* = 12.1, 1.8 Hz, H-6″), 3.63 (1H, m, H-6″), 3.63 (3H, S, H-12), 3.21-3.35 (4H, m, H-2*″*,3*″*,4*″*,5*″*), 3.07 (1H, t, *J* = 7.0 Hz, H-9).

**FIGURE 1:**
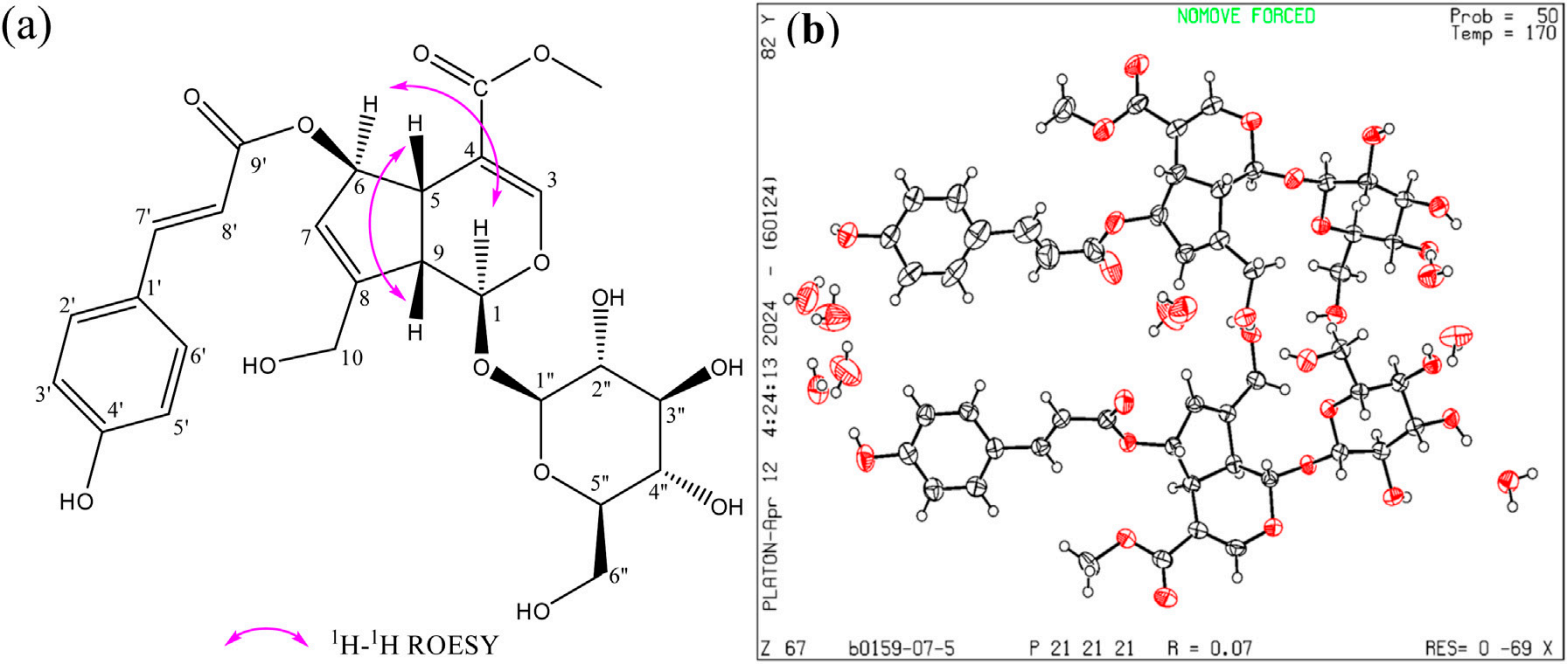
(1*S,*5*S,*6*R,*9*S*)-(*E*)-6-*O*-(*p*-coumaroyl) scandoside methyl ester (C_26_H_30_O_13_); CAS No: 83946-90-1; **(a)** Key ROESY correlations; **(b)** X-ray structure of (*E*)*-*6-*O-*(*p*-coumaroyl) scandoside methyl ester showing the absolute configuration.

^13^C NMR (125 MHz, Methanol-*d*_4_); *δ* 169.2 (C-11), 169.0(C-9′), 161.4 (C-4′), 154.2 (C-3), 150.5 (C-8), 146.7 (C-7′), 131.3 (C-2′, 6′), 127.4 (C-1′), 127.3 (C-7), 117.0 (C-3′, 5′), 115.6 (C-8′), 110.1 (C-4), 100.4 (C-1″), 98.1 (C-1), 83.8 (C-6), 78.6 (C-3″), 78.0 (C-5″), 74.9 (C-2″), 71.7 (C-4″), 62.8 (C-6″), 61.1 (C-10), 52.2 (C-12), 47.0 (C-9), 42.4 (C-5). The relative configuration of (*E*)-6-*O*-(*p*-coumaroyl) scandoside methyl ester was assigned by ROESY experiment. The ROESY correlations H-C (5)/H-C (9) and H-C (1)/H-C (6) provided the relative configurations of the stereogenic centers of (*E*)-6-*O*-(*p*-coumaroyl) scandoside methyl ester (Figure 1a). Furthermore, the absolute configuration of (*E*)-6-*O*-(*p*-coumaroyl) scandoside methyl ester was established as 1*S*,5*S*,6*R*,9*S* by X-ray diffraction analysis according to Flack parameter, using anomalous dispersion with copper radiation (Figure 1b). Taken together, the structure of (*E*)-6-*O*-(*p*-coumaroyl) scandoside methyl ester was elucidated as depicted in Figure 1.

### X-ray crystallographic analysis of (*E*)-6-*O*-(*p*-coumaroyl) scandoside methyl ester

C_26_H_30_O_13_, MW = 550.51, orthorhombic, Space group P2 (1)2 (1)2 (1), with lattice parameters *a* = 8.53837 (19) Å, *b* = 15.6644 (4) Å, *c* = 44.4866 (13) Å, resulting in a volume (V) of 5950.0 (3) Å^3^, *Z* = 8. The calculated density was 1.405 g/cm^3^, with a temperature (T) of 169.99 (10) K, and a linear absorption coefficient (Cu Kα) of 1.028 mm^-1^, *F* (000) = 2670.0. A colorless crystal of dimensions 0.15 × 0.12 × 0.11 mm was selected for X-ray analysis. A total of 44828 reflections, collected in the range 5.982° ≤ 2*θ* ≤ 148.07°, yielded 11799 unique reflections. The crystal was kept at 169.99 (10) K during data collection. Using Olex2, the structure was solved with the SHELXT structure solution program using Intrinsic Phasing and refined with the SHELXL refinement package using Least Squares minimization. Hydrogen atoms were fixed at calculated positions. The final indices were *R*_1_ = 0.0669 (*I* > 2*σ*(*I*)) and *wR*_2_ = 0.1742 with Goodness-of-fit on F^2^ values for 1.019.

### Development of quality standard for *Scleromitrion diffusum* by HPLC-DAD

In this study, a green and efficient chromatographic method was established based on the quality chemical marker, (*E*)-6-*O*-(*p*-coumaroyl) scandoside methyl ester. Sample solution preparation, HPLC system, column and injection volume were consistent with chromatographic fingerprint method. The separations were optimized at 40°C, with a flow rate of 1 mL/min, using a mobile phase of methanol/water (35:65, v/v) in isocratic elution mode, and detection at wavelength of 313 nm.

The quality marker standard stock solution at a concentration of 3000 μg/mL was prepared in methanol and stored at 4°C. Calibration curves were established with five concentrations within the range of 30.5-762.5 μg/mL by appropriate dilution of the stock solution with methanol. A reference solution (in methanol) at concentration of 300 μg/mL was prepared. The proposed HPLC method for quality marker quantitative analysis was validated by determining the linearity, repeatability, stability, intra-day, and inter-day precision.

## Results and discussion

### Establishment of TLC chromatographic fingerprint of *Scleromitrion diffusum* and related species

**FIGURE 2:**
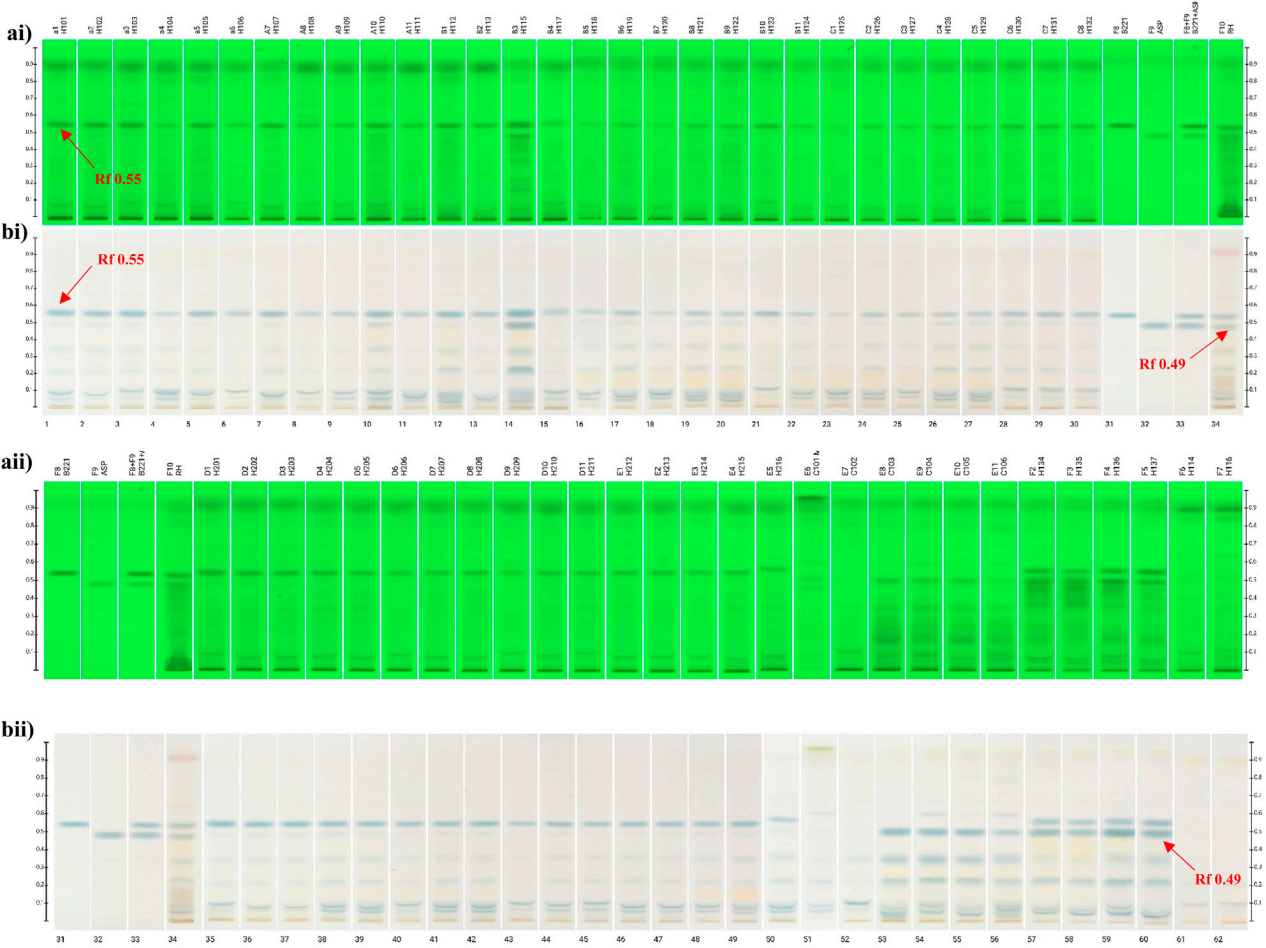
TLC fingerprinting of *Scleromitrion diffusum, Hedyotis corymbosa*, *Hedyotis tenelliflora,* reference markers and standard herb. **(ai,aii)** UV 254 nm prior derivatization; **(bi,bii)** Observed under visible light after spraying with 10% sulphuric acid in ethanol; *Scleromitrion diffusum* herb materials (tracks 1–30); self-manufacture (*E*)-6-*O*-(*p*-coumaroyl)scandoside methyl ester (track 31); Asperuloside (track 32); reference mixture (track 33); standard herb (track 34); *Scleromitrion diffusum* decoction pieces (tracks 35–50); *Hedyotis corymbosa* herb materials (tracks 51–52); fresh *Hedyotis corymbosa* (tracks 53–56); fresh *Scleromitrion diffusum* (tracks 57–60); *Hedyotis tenelliflora* (tracks 61–62).

The TLC fingerprint chromatograms of both *S. diffusum* herbal materials and decoction pieces exhibited no significant variations under UV 254 nm (Figures 2ai, 2aii). Notably, all the *S. diffusum* samples (Tracks 1-46) including herbal materials and decoction pieces exhibited a distinct fluorescence absorption band at *R*_*f*_ 0.55 in the chromatographic fingerprint, consistent with the standard herb (Track 34). However, this absorption band was not observed on the tracks 51 - 56 and tracks 61–62, which belong to its confusing species *H. corymbosa* and *H. tenelliflora,* respectively. Furthermore, after the TLC plate was sprayed with 10% solution of sulfuric acid in ethanol and heated on a TLC plate heater at approximately 105°C, the colors of such bands appeared distinctly. Meanwhile, a noticeable blue band was observed at the same position (R_*f*_ value 0.55) in all *S. diffusum* samples under visible light. However, this distinctive band was conspicuously absent in all samples of *H. corymbosa* and *H. tenelliflora* (Figures 2aii, 2bii). This observation implies that this component at R_*f*_ value 0.55 with a significant fluorescence absorption was unique to *S. diffusum* and may serve as a characteristic identifying chemical marker. Thus, this significant disparity in chemical composition among the three distinct species can be leveraged to differentiate *S. diffusum* from its common adulterants using a straightforward TLC method.

Next, to elucidate the composition of this compound and investigate its fundamental chemical properties, a series of column chromatographic separations of the 70% ethanol extract from *S. diffusum* was conducted following the TLC-guided isolation method (Supplementary Figure S1). Finally, this component was isolated and identified as (1*S,*5*S,*6*R,*9*S*)-(*E*)-6-*O*-(*p*-coumaroyl) scandoside methyl ester (CAS No: 83946-90-1) basing on comparison with spectral data available in the literature, including ^1^H-NMR, ^13^C-NMR, DEPT, 2D-NMR, LC-HRESIMS and X-ray (Park and Whang, 2020; Nishihama et al., 1981; Xu et al., 2010; Wu et al., 1991). We then conducted a systematic search on this compound using electronic databases including SciFinder, PubMed, Science Direct, Google Scholar, and China National Knowledge Infrastructure (CNKI), using the term “83946-90-1” and “(*E*)-6-*O*-(*p*-coumaroyl) scandoside methyl ester,” and the results showed that this chemical marker is a specific chemical component of *S. diffusum* and was not found in any other herbs. Furthermore, some previous studies have demonstrated that (*E*)-6-*O*-(*p*-coumaroyl) scandoside methyl ester can be used for the quality control of *S. diffusum* (Li et al., 2010; Wang et al., 2018; Zhang et al., 2008; Yang et al., 2015). Our discovery suggests that this component holds promise as a potential chemical marker for the identification and quality control of *S. diffusum*.

**FIGURE 3:**
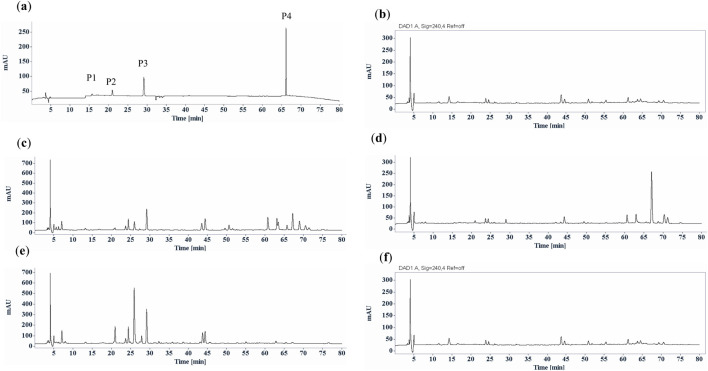
Typical HPLC chromatographic profiles (UV chromatograms at 254 nm) of *Scleromitrion diffusum,* **(H)** *corymbosa* and *Hedyotis tenelliflora.* **(a)** reference mixture, deacetyl asperulosidic acid methyl ester (P1), scandoside methyl ester (P2), asperuloside (P3) and (*E*)-6-*O*-(*p*-coumaroyl) scandoside methyl ester (P4); **(b)** *Hedyotis tenelliflora*; **(c)** fresh *Scleromitrion diffusum*; **(d)** *Scleromitrion diffusum* herbal materials; **(e)** fresh *H*. *corymbosa*; **(f)** *H*. *corymbosa* herbal materials.

As a qualified pharmacopoeial marker, this component must exhibit specific characteristics, with strict requirements for its content in traditional Chinese medicine. Trace ingredients in plants for identification and quality control should be avoided. Therefore, quantification of this compound using HPLC analysis was also conducted. The typical HPLC profiles of *S. diffusum*, *H corymbosa* and *H. tenelliflora* confirmed that the potential chemical marker was only found in *S. diffusum* with retention time at 67 min, but totally absent in *H*. *corymbosa* and *H. tenelliflora* (Figure 3; Table 1)*.* From Table 1, the contents of the chemical marker in *S. diffusum* were 0.44% ± 0.17% in herbal materials (*n* = 6) and 0.51% ± 0.12% in fresh herb (*n* = 4), respectively, which were of no significant difference (*p* = 0.45) among different types and different sources of *S. diffusum,* complying with the quality control requirements. Based on the existence of this compound, it can be fairly straightforward to differentiate *S. diffusum* from substitutes like *H*. *corymbosa* and *H. tenelliflora*, also corroborating the experimental conclusions of TLC. Furthermore, these findings indicated that this compound was a better representative chemical marker for the identification and quality control than triterpenes (oleanolic acid, ursolic acid), phenolic acid (*p*-coumaric acid, ferulic acid), flavonoids (rutin, quercetin, kaempferol), and aesculetin, which are commonly found in herbal medicines. However, some studies have shown that *H. corymbosa* contains trace amounts of (*E*)-6-*O*-(*p*-coumaroyl) scandoside methyl ester (Wang et al., 2018; Otsuka et al., 1991). Nevertheless, our TLC and HPLC analysis results indicated that (*E*)-6-*O*-(*p*-coumaroyl) scandoside methyl ester was undetectable in *H. corymbosa*. This discrepancy may be due to the contamination of *H. corymbosa* samples with a small amount of *S. diffusum*.

**TABLE 1:** Quantitative results of certain chemical components present in *Scleromitrion diffusum*, *Hedyotis corymbosa* and *Hedyotis tenelliflora*.

No.	Scandoside methyl ester (%)	Asperuloside (%)	Proposed marker^a^(%)	Species	Type
H101	0.05	0.03	0.51	*Scleromitrion diffusum* (Willd.) R. J. Wang	Commercial Medicinal materials
H102	0.04	0.05	0.54	*Scleromitrion diffusum* (Willd.) R. J. Wang	
H103	0.21	0.03	0.60	*Scleromitrion diffusum* (Willd.) R. J. Wang	
H104	0.03	0.01	0.16	*Scleromitrion diffusum* (Willd.) R. J. Wang	
H105	0.02	0.02	0.50	*Scleromitrion diffusum* (Willd.) R. J. Wang	
H106	0.02	0.01	0.32	*Scleromitrion diffusum* (Willd.) R. J. Wang	
mean ± SD	0.06 ± 0.07	0.025 ± 0.02	0.44 ± 0.17		
H134	0.17	1.06	0.46	*Scleromitrion diffusum* (Willd.) R. J. Wang	Fresh plants dried in the lab
H135	0.21	0.56	0.41	*Scleromitrion diffusum* (Willd.) R. J. Wang	
H136	0.24	2.26	0.54	*Scleromitrion diffusum* (Willd.) R. J. Wang	
H137	0.48	1.11	0.69	*Scleromitrion diffusum* (Willd.) R. J. Wang	
mean ± SD	0.28 ± 0.14	1.25 ± 0.72	0.53 + 0.12		
HC101	0.33	0.02	—	*Hedyotis corymbosa* (L.) Lam	Commercial Medicinal materials
HC102	0.13	0.01	—	*Hedyotis corymbosa* (L.) Lam	
HC107	0.18	0.06	—	*Hedyotis corymbosa* (L.) Lam	
HC108	0.03	0.11	—	*Hedyotis corymbosa* (L.) Lam	
mean ± SD	0.17 ± 0.13	0.05 ± 0.05	—		
HC103	1.34	0.83	—	*Hedyotis corymbosa* (L.) Lam	Fresh plants dried in the lab
HC104	1.74	0.66	—	*Hedyotis corymbosa* (L.) Lam	
HC105	1.35	0.64	—	*Hedyotis corymbosa* (L.) Lam	
HC106	0.70	0.23	—	*Hedyotis corymbosa* (L.) Lam	
mean ± SD	1.28 ± 0.43	0.59 ± 0.25			
H114	—	—	—	*Hedyotis tenelliflora* Blume	Commercial Medicinal materials
H116	—	—	—	*Hedyotis tenelliflora* Blume	

- Below detection limit.

^a^ (*E*)-6-*O*-(*p*-coumaroyl) scandoside methyl ester.

### Advantages over asperuloside, deacetyl asperulosidic acid methyl ester, and scandoside methyl ester as chemical markers for *Scleromitrion diffusum*

A large number of literature reports that the compound asperuloside can be used as a chemical marker for the identification and quality control of *S. diffusum* (Wang et al., 2018; Yang et al., 2015; Zhai and Lv, 2016), This component has also been formally adopted as a chemical marker in the Hong Kong Chinese Materia Medica Standards and the Taiwan Pharmacopoeia. Interestingly, both of these herbal medicine standards have established the same quantitative limit for asperuloside, i.e., the herbal sample should contain not less than 0.09% of asperuloside (C_18_H_22_O_11_), calculated with reference to the dried substance.

As shown in Figure 2, our TLC fingerprint analysis results also revealed another distinct blue band of asperuloside under the band of (*E*)-6-*O*-(*p*-coumaroyl) scandoside methyl ester, with a *R*_*f*_ value of 0.49 appears in the reference (Track 32), standard herb (Track 34) and fresh herbs, including four batches of fresh *S. diffusum* (Tracks 57-60). However, unexpectedly, four batches of fresh *H. corymbosa* (Tracks 53-56) also displayed the same distinct blue band at the same position. In contrast, among the 30 batches of *S. diffusum* herbal materials and 16 batches of *S. diffusum* decoction pieces, only two batches of *S. diffusum* herb materials (Tracks 10 and 14) exhibited a distinct blue band of asperuloside. Further TLC detailed analysis revealed that the band of asperuloside was also detected in almost all the samples of *S. diffusum*, but the intensity of asperuloside band is significantly lighter than fresh *S. diffusum* herbal materials (Tracks 57-60) and fresh *H. corymbosa* herbal materials (Tracks 53–56). This blue band was undetectable in two batches *H. tenelliflora* herbal materials (Tracks 61–62).

Similarly, this significant difference in the TLC fingerprint analysis was quantitatively reflected in the HPLC analysis. Our HPLC results also revealed a substantial amount of asperuloside in four batches of fresh *S. diffusum*, ranging from 0.56% to 2.26%, which is significantly higher than the 0.09% minimum content limitation specified in the Hong Kong Chinese Materia Medica Standards and the Taiwan Pharmacopoeia. However, according to the HPLC content measurements (Table 1), the content of asperuloside in all *S. diffusum* herbal materials and decoction pieces (samples 1-30, 35-50) was no more than 0.05%, which is considerably lower than the general recommended minimum content requirement of the Chinese Pharmacopoeia (more than 0.1%). Generally, the main constituents in herbal medicines are responsible for their therapeutic effects. Therefore, it is advisable to avoid selecting components present in very low or trace amounts in herbal medicines as chemical markers for identification and quality control. What is even worse is that the content of asperuloside in the common adulterants of four batches of fresh *H. corymbosa* and four batches of *H. corymbosa* herbal materials was 0.59% ± 0.22% (n = 4) and 0.05% ± 0.04% (*n* = 4), respectively. These values of asperuloside in fresh *H. corymbosa* were approximately 20 times higher than those in *S. diffusum* herbal materials which was only 0.025 ± 0.02 (*n* = 6). In summary, asperuloside is not a specific chemical marker for *S. diffusum* as it is also present in *H. corymbosa*, especially in fresh samples. This discrepancy leads to a challenge in the identification and quality control of *S. diffusum* using the methods outlined in the Hong Kong Chinese Materia Medica Standards and the Taiwan Pharmacopoeia, because it is impossible to differentiate *H. corymbosa*, especially considering its superior quality in fresh *H. corymbosa* as compared to *S. diffusum* herbal materials.

**FIGURE 4:**
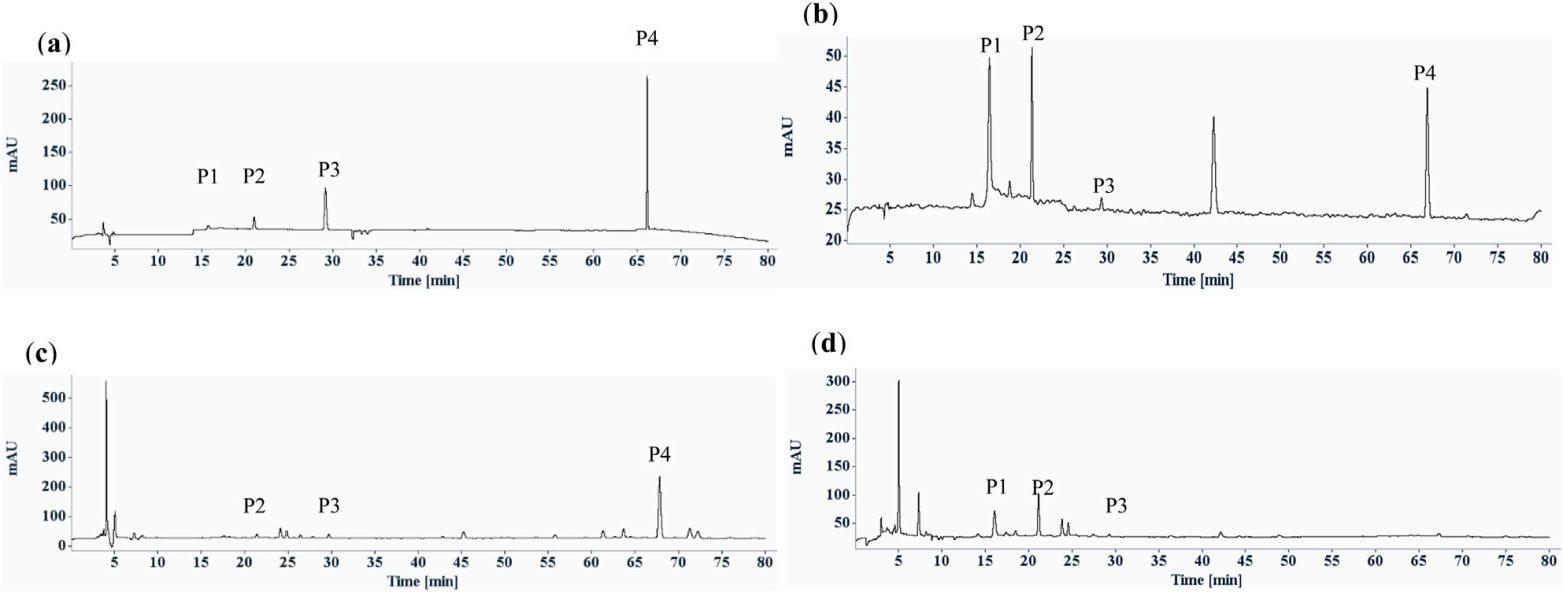
Typical HPLC chromatographic profiles (UV chromatograms at 254 nm) of *Scleromitrion diffusum*. **(a)** reference mixture; deacetyl asperulosidic acid methyl ester (P1), scandoside methyl ester (P2), asperuloside (P3) and (*E*)-6-*O*-(*p*-coumaroyl) scandoside methyl ester (P4); **(b)** The heat denaturation of the purified (*E*)-6-*O*-(*p*-coumaroyl) scandoside methyl with 100°C heating for 60 min; **(c)** *Scleromitrion diffusum* herbal materials refluxed with 70% EtOH; **(D)** *Scleromitrion diffusum* herbal materials refluxed with water.

This significant disparity in the chemical composition and content between fresh and dried materials may be due to different drying methods. In this study, all the *S. diffusum* herbal materials and decoction pieces sourced from different herbal markets or traditional Chinese medicine companies were sun-dried. Conversely, the four batches of fresh *S. diffusum* and four batches of fresh *H. corymbosa*, collected concurrently in Hong Kong, were immediately oven-dried at 55°C for 24 h. As shown in Figures 3C–F, compared to the oven-dried fresh *H. corymbosa*, not only did the asperuloside content significantly decrease, but the content of most other components in the *H. corymbosa* herbal materials also showed a noticeable reduction. This phenomenon indicates that variations in drying methods alone can yield substantial differences in the content of herbal medicine ingredients. In light of these findings, the use of asperuloside, as a chemical marker for identification and quality control of *S. diffusum* may be flawed due to its unstable structure. To ensure the consistency and efficacy of *S. diffusum*, comprehensive research and standardized drying methods are imperative. But so far, there remains a significant gap in the research on the impact of different drying methods on the chemical composition and efficacy of *S. diffusum*. But for the (*E*)-6-*O*-(*p*-coumaroyl) scandoside methyl ester, fortunately there were no significant effect between different drying methods according to the HPLC analysis results (Table 1; Figures 3, 4).

HPLC and TLC are the most popular methods for identifying and controlling the quality of traditional Chinese medicines, and extensively adopted by the Chinese Pharmacopoeia. Statistics show that the percentage of HPLC and TLC chromatographic methods applied in 2015 edition of the Chinese Pharmacopoeia reached 51.19% and 42.58%, respectively (Shen et al., 2021). However, for traditional Chinese medicines like *S. diffusum* listed only in the Appendix of the Chinese Pharmacopoeia, standardized methods for chemical identification and quality control are still lacking. Once the chemical marker for identification and quality control was determined, we have investigated and established a comprehensive HPLC quantification method for the determination of this marker in *S. diffusum* from different origins according to the pharmacopoeia standards.

On the other hand, many studies have also highlighted the potential use of deacetyl asperulosidic acid methyl ester and its structural analog scandoside methyl ester for the quality control of *S. diffusum* (Park and Whang, 2020; Zhang et al., 2008; Yang et al., 2015; Zhai and Lv, 2016). However, our HPLC results indicated that deacetyl asperulosidic acid methyl ester was not detected in the three species. The content of scandoside methyl ester in commercial medicinal materials was only 0.06% ± 0.07% and far less than in fresh *S. diffusum* herbal materials dried in the lab. As shown in Table 1 *,* similar to asperuloside, the content of scandoside methyl ester in fresh *H. corymbosa* herbal materials on average is 1.28% ± 0.43% and even exceeded the content in *S. diffusum* decoction pieces. Therefore, scandoside methyl ester is not a specific chemical marker that can distinguish *S. diffusum* from counterfeits. What is even worse is that this compound was also present in higher amounts in *Gardenia* species (Liu et al., 2012).

In the Chinese Pharmacopoeia 2020, *S. diffusum* is also a major ingredient in various Chinese herbal formulations such as Huahong tablets, Huahong capsules, Huahong granules, with deacetyl asperulosidic acid methyl ester as the designated chemical marker for quality control (National Pharmacopoeia Committee, 2020). Structural analysis of this compound reveals that it is formed by the esterification of one molecule of *p*-coumaric acid and one molecule of scandoside methyl ester, and it contains a glycosidic bond, classifying it as an iridoid glycoside. Due to the presence of glycosidic and ester bonds in the structure, this chemical marker exhibits chemical instability under certain conditions, making it susceptible to hydrolysis reactions. As shown in Figure 4B, examination of the stability of the purified (*E*)-6-*O*-(*p*-coumaroyl) scandoside methyl ester in boiling water revealed that this chemical marker is unstable, gradually hydrolyzing to produce *p-*coumaric, deacetyl asperulosidic acid methyl ester and scandoside methyl ester. Considering that these Chinese herbal formulations all use boiling water extraction, (*E*)-6-*O*-(*p*-coumaroyl) scandoside methyl ester would hydrolyze to deacetyl asperulosidic acid methyl ester during the boiling water extraction process. As shown in Figures 4C,D, compared to the ethanol extract of *S. diffusum*, (*E*)-6-O-(p-coumaroyl) scandoside methyl ester almost completely disappears in the water extract of *S. diffusum*, with a corresponding significant increase in the hydrolysis product deacetyl asperulosidic acid methyl ester. Therefore, it is reasonable for the pharmacopoeia formulations to use deacetyl asperulosidic acid methyl ester as the chemical marker. However, it is advisable to avoid using decomposition or degradation products as chemical markers for the identification and quality control of single herbal medicines.

Consequently, the prototype component (*E*)-6-*O*-(*p*-coumaroyl) scandoside methyl ester of *S. diffusum* was selected as the chemical marker for identification and quality control to avoid selecting trace components or degradation products, in accordance with pharmacopoeial requirements. The extraction efficiency was optimized by changing the extraction methods, solvent composition, solvent ratio, and extraction time. Among the different approaches tested, extraction with 70% ethanol and refluxing in a water bath for 80 min yielded the highest marker content. Furthermore, the optimized green and efficient chromatographic method for quality marker was validated. As shown in Table 2, the calibration curves showed remarkable linearity (R^2^ > 0.9990) within the test ranges. The relative standard deviation (RSD) values of intra-day and inter-day variations, repeatability, and stability of the (*E*)-6-*O*-(*p*-coumaroyl) scandoside methyl ester was less than 1.86%. The overall recovery (calculated using sample solution of known content spiked with 80%, 100%, and 120% standard solutions) ranged between 90.49 and 92.20. According to the optimized and validated chromatographic method, 29 batches of commercial medicinal materials and 16 batches of decoction pieces were quantified (*n* = 4). As the results were shown in the Supplementary Tables S3, S4, the content of quality chemical marker in commercial medicinal materials was 0.31% ± 0.13%, ranging from 0.10%–-0.56% and in decoction pieces was 0.33% ± 0.08%, ranging from 0.19%-0.54%. The content limitation of (*E*)-6-*O*-(*p*-coumaroyl) scandoside methyl ester could be set at no less than 0.20% as the quality standard, according to 35% reduction of the mean content in those commercial medicinal materials tested.

**TABLE 2:** Validation of chromatographic method for quality standard.

Standard curve^a^	*R* ^*2*^	Linear range (μg/mL)	Precision (RSD, %)^b^		Repeat-ability^c^ (RSD, %, *n* = 6)	Stability^d^ (RSD, %, *n* = 7)	Recovery^f^ (%, *n* = 3)
			Intra-day (*n* = 6)	Inter-day (*n* = 3)			
*y* = 13508*x* - 35.434	0.9995	30.5–762.5	0.56	1.86	1.49	0.28	90.49
							91.82
							92.20

^a^ Standard curves: *y* is the area of the characteristic absorption peak; *x* is the concentration of an authentic standard in extract solution; LOD, limit of detection (signal-to-noise ratios (*S*/*N*) = 3); LOQ, limit of quantitation (*S*/*N* = 10).

^b^ RSD (%) of intra-day precision = (SD, of amount detected/mean of amount detected) × 100%.

RSD (%) of inter-day precision = the RSD, in three consecutive days.

^c^ Repeatability (%) = the RSD, of the content of six replicates of the sample.

^d^ Stability (%) = the RSD, of the sample analyzed at 0, 1, 2, 4, 8, 12, and 24 h.

^e^ Recovery (%) = 100% × (amount detected–original amount)/amount spiked.

At the same time, the TLC identification method was also optimized and established, including different TLC plates, development systems, and ratio optimization, as well as the derivatization reagents and observation conditions. Eventually, it was determined that the same extraction method used in HPLC content determination could be employed, with silica gel GF_254_ as the thin layer stationary phase, and the mobile phase was ethyl acetate: methanol: water = 8:1.5:0.7. The 10% v/v sulfuric acid in ethanol was used as the derivatization reagent. Additionally, the applicability of the developing system was also investigated including various brands of thin layer plates, different temperatures and humidity conditions.

According to the “Technical Requirements for the Raw Materials and Documentation of Traditional Chinese Medicine Chemical Reference Standards”, the chemical marker should be suitable for large-scale preparation with a purity not less than 98%. Since this chemical marker (*E*)-6-*O*-(*p*-coumaroyl) scandoside methyl ester is exclusive to *S. diffusum*, we endeavored to establish a laboratory process for extracting, isolating, and purifying this chemical marker on a large-scale basis from *S. diffusum*. To start off, two small preliminary experiments were conducted to optimize the separation process and determine the laboratory preparation procedure for this compound. As shown in Supplementary Figure S1, through a series of different column chromatography, 20 g of chemical marker were isolated from 10 kg of *S. diffusum* with a yield of 0.2%. During this purification process, it was observed that the purity of the chemical marker obtained through preparative HPLC was still insufficient (less than 98% w/w). However, we found that under certain conditions, this compound is easy to be crystallized. Consequently, the compound was recrystallized ten times until no impurities were detected in the mother liquor, and subsequent HPLC purity analysis revealed that the purity of this compound then exceeded 99% w/w, even exceeded that of the commercially available chemical marker. The basic information of this chemical marker are as follows: Hygroscopic, at 8.7% after absorbing moisture; Residue on ignition: 0.16%; Water content: 3.7%; Residual of Methanol: None detected (limit of detection: 0.003 g/100 g); Content of salt-forming (sodium ions and potassium ions): None detected (limit of detection: 3 mg/kg). Since chemical stability is a critical factor in determining the suitability of a compound as a chemical marker, preliminary stability tests were conducted, including high-temperature and high-humidity experiments. Our TLC results demonstrated that this potential chemical marker is stable under high temperature and high humidity conditions, with no noticeable metabolites detected. Light exposure test, oxidation test, and both accelerated and long-term stability tests should be performed in the future.

## Conclusion

In this study, 58 samples of either commercial or freshly collected *S. diffusum* and its confusing species were analyzed by TLC and HPLC. The results showed that the known chemical marker asperuloside is only present in very low concentrations in *S. diffusum* herbal materials and decoction pieces, but in high concentrations in fresh *H. corymbosa* herbal materials, making it unsuitable as a chemical marker compound for *S. diffusum*. Through TLC and HPLC analysis, a specific chemical marker suitable for identification and quality control was identified as (*E*)-6-*O*-(*p*-coumaroyl) scandoside methyl ester, which is specific to *S. diffusum*.

Based on the chemical properties of this chemical marker and the required standards of the Chinese Pharmacopoeia, we have developed simple, fast, highly selective and standardized TLC identification and HPLC analysis and quantitative method, and have successfully applied this method for identification and quality control of *Scleromitrion difffusum* and its substitutes. Although the various components in herbal medicines make quality control more complex, the introduction of this chemical marker addresses the challenges in the identification and quality control of *S. diffusum*. On the other hand, previous studies have reported that this chemical marker exhibits strong inhibition of the formation of advanced glycation end-product (AGE) and butyrylcholinesterase (BChE) for the treatment of Alzheimer’s disease, and showed a protective effect on LPS-induced renal inflammation in mice, and exhibited moderate anti-proliferation effect on PC3 human androgen-independent prostate cancer cells (Park and Whang, 2020; Li et al., 2010; Liu et al., 2006; Ye et al., 2015). Now with our establishment of the laboratory-scale preparation method for this compound, and with more biological studies (in particular on the potential anti-cancer effect) (Lau, 2014) on this compound in the near future, it is anticipated that this proposed chemical marker may be eventually upgraded to a biological marker for *S. diffusum*.

In conclusion, we reported for the first time that the compound (*E*)-6-*O*-(*p*-coumaroyl) scandoside methyl ester which is specific to *S. diffusum*, could serve as the chemical marker. Our findings have provided valuable insights to the identification and quality control processes for *S. diffusum*, and anticipated with potential applications by the industry and regulatory authority. It is hoped that our proposal of this new chemical marker for *S. diffusum* will be considered by the future editions of the Chinese Pharmacopoeia and other pharmacopoeias.

## Data Availability

The original contributions presented in the study are included in the article/Supplementary Material, further inquiries can be directed to the corresponding authors.
