# Supplementary material for: Proposal of a new chemical marker for the quality control of the herb Scleromitrion diffusum
Source: Front Chem. 2025 Apr 29;13:1600769. doi: 10.3389/fchem.2025.1600769 (PMC12069056; doi:10.3389/fchem.2025.1600769)
Supplement: Supplementary file 1 [file DataSheet1.docx]

Proposal of a new chemical marker for the quality control of the herb *Scleromitrion diffusum*

Tao Zheng^a,b,#^, Chu-Chu Zhong^c,d,#^, Grace Gar-Lee Yue^e^, Dong-Min Jiang^c^, Man-Ho Tong^a,b^, Hiu-Yan Wong^f^, Kwan-Ho Wong^d,f,g^, Meng-Hua Wu^c^, David Tai-Wai Lau^f,^*, Hui Cao^c,^*, Pang-Chui Shaw^a,b,d,g,^*, Clara Bik-San Lau^a,e,h,^*

^a^ Institute of Chinese Medicine, The Chinese University of Hong Kong, Shatin, New Territories, Hong Kong SAR, China;

^b^ State Key Laboratory of Research on Bioactivities and Clinical Applications of Medicinal Plants, The Chinese University of Hong Kong, Shatin, New Territories, Hong Kong SAR, China;

^c^ College of Pharmacy, Jinan University, Guangzhou 510632, China;

^d^ Li Dak Sum Yip Yio Chin R & D Centre for Chinese Medicine, The Chinese University of Hong Kong, Shatin, New Territories, Hong Kong SAR, China;

^e^ Department of Pharmacology and Pharmacy, LKS Faculty of Medicine, The University of Hong Kong, Pokfulam, Hong Kong SAR, China;

^f^ Shiu-Ying Hu Herbarium, School of Life Sciences, The Chinese University of Hong Kong, Shatin, New Territories, Hong Kong SAR, China;

^g^ School of Life Sciences, The Chinese University of Hong Kong, Shatin, New Territories, Hong Kong SAR, China;

^h^ School of Chinese Medicine, Li Ka Shing Faculty of Medicine, The University of Hong Kong, Pokfulam, Hong Kong SAR, China.

^#^ The authors contributed equally.

*Corresponding authors:

Clara Bik-San Lau, Department of Pharmacology and Pharmacy, LKS Faculty of Medicine

The University of Hong Kong, Hong Kong SAR, China.

Email: [cbslau@hku.hk](mailto:cbslau@hku.hk)

Pang-Chui Shaw, School of Life Sciences, Li Dak Sum Yip Yio Chin R & D Centre for Chinese Medicine, Institute of Chinese Medicine & State Key Laboratory of Research on Bioactivities and Clinical Applications of Medicinal Plants, The Chinese University of Hong Kong, Shatin, New Territories, Hong Kong SAR, China.

Email: [pcshaw@cuhk.edu.hk](mailto:pcshaw@cuhk.edu.hk)

Hui Cao, College of Pharmacy, Jinan University, Guangzhou, China

Email: [kovhuicao@aliyun.com](mailto:kovhuicao@aliyun.com)

David Tai-Wai Lau, Shiu-Ying Hu Herbarium, School of Life Sciences, The Chinese University of Hong Kong, Shatin, New Territories, Hong Kong SAR, China.

Email: [lautaiwai@cuhk.edu.hk](mailto:lautaiwai@cuhk.edu.hk)

**Supplementary Information**

**Figure S1.** Flow diagram of extraction, isolation and purification of the chemical marker (*E*)*-*6-*O-*(*p*-coumaroyl) scandoside methyl ester from *S. diffusum*

Recrystallization for 10 times (Methanol: H_2_O = 1:5)

6 times

5 times

Subjected to column chromatography on RP-18 using 23 % ACN （Preparative HPLC）

Subjected to column chromatograph on silica gel (230-400 mesh) using ethyl acetate–methanol = 1:0 and 12:1, 8:1, v/v，0.9 L)

Extraction with 70 % EtOH (10 × 1h × 3 times) under reflux

Sephadex LH-20 using Methanol, 2L

Subjected to column chromatography on D101 column using 20 % EtOH, 7L; 60 % EtOH, 7L

Diluted the condensate with distilled water

Liquid-liquid Extraction (hexane, *n*-butanol)

*S. diffusum* (2.4kg)

Ethanol extract

N-hexane fraction

Water fraction

N-butanol fraction 170g

Fr. B1

0-32 bottle

Fr. B3

Fr. B2 (60 % Ethanol fraction 25g)

Fr. B21 (Negative)

Fr. B221

Fr. B23

Fr. B22

Fr. B222 (8:1 fraction 8g)

Fr. B223

(*E*)-6-O-(*p*-coumaroyl) scandoside methyl ester

(*E*)-6-O-(*p*-coumaroyl) scandoside methyl ester crystal

**a**

**b**

**C**

**d**

**e**

**f**


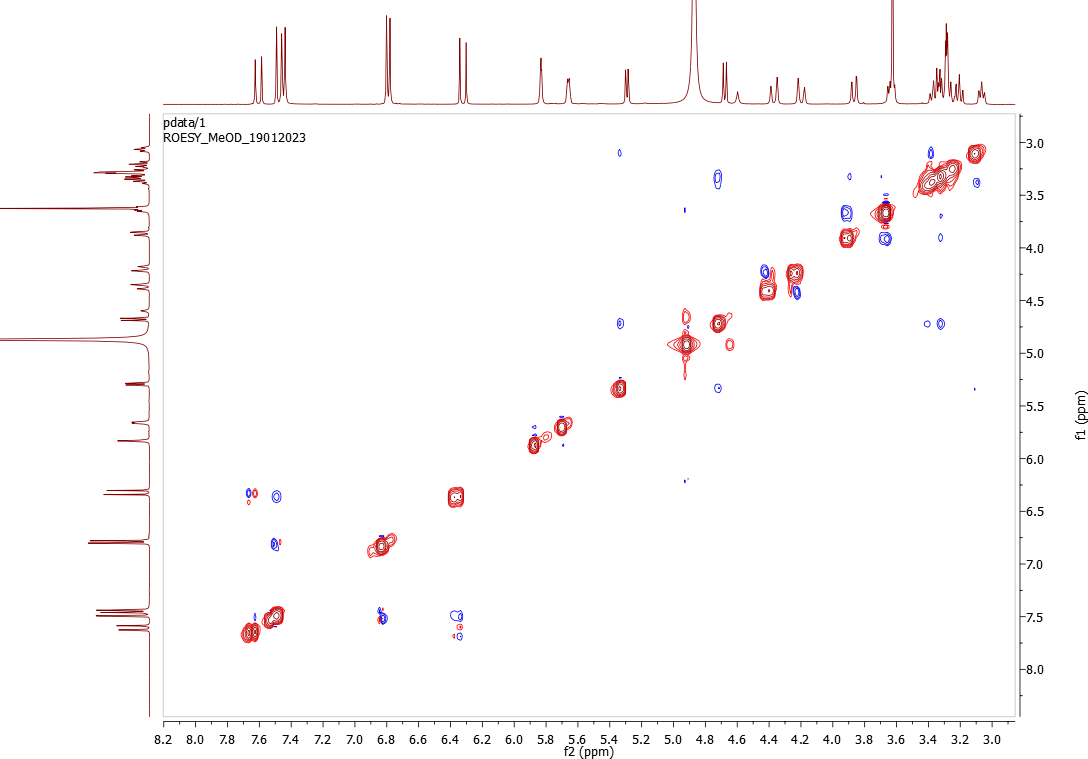


**Figure S2.** NMR spectra (MeOH-*d*_4_, 500 MHz) of (*E-*6-*O-*(*p*-coumaroyl scandoside methyl ester. **a)** ^1^H NMR spectra; **b)** ^13^C and DEPT spectra (MeOH-*d*_4_, 125 MHz); **c)** HSQC spectra (CDCl_3_, 500 MHz); **d)** HMBC spectra (MeOH-*d*_4_, 500 MHz); **e)** ^1^H-^1^H COSY; **f)** ^1^H-^1^H ROESY.

**Table S1.** Information of herbal materials and chemical components used in this study

| **No.** | **Collector Number** | **Authenticated species** | **Province/ Source** | **Classification** | **batch number** | **Quantity (KG)** | **Collecting time** |
| --- | --- | --- | --- | --- | --- | --- | --- |
|  | H101 | *Scleromitrion diffusum* | Jiangxi | wild | 202111027 | 0.5 | 2022.09.07 |
|  | H102 | *Scleromitrion diffusum* | Guangdong | wild | Y02132-22001 | 0.5 | 2022.09.07 |
|  | H103 | *Scleromitrion diffusum* | Fujian | wild | WLY22E01 | 0.5 | 2022.09.07 |
|  | H104 | *Scleromitrion diffusum* | Guangdong | wild | NO | 0.5 | 2022.09.07 |
|  | H105 | *Scleromitrion diffusum* | Guangxi | cultivated | NO | 0.5 | 2022.09.07 |
|  | H106 | *Scleromitrion diffusum* | Hubei | wild | NO | 0.5 | 2022.09.07 |
|  | H107 | *Scleromitrion diffusum* | Jiangxi | wild | NO | 0.5 | 2022.09.07 |
|  | H108 | *Scleromitrion diffusum* | Henan | wild | NO | 0.5 | 2022.09.07 |
|  | H109 | *Scleromitrion diffusum* | Henan | cultivated | 20220902 | 0.1 | 2022.09.07 |
|  | H110 | *Scleromitrion diffusum* | Hunan | cultivated | 20220903 | 0.3 | 2022.10.11 |
|  | H111 | *Scleromitrion diffusum* | Hubei | wild | NO | 0.45 | 2022.10.11 |
|  | H112 | *Scleromitrion diffusum* | Jiangxi | wild | NO | 0.3 | 2022.10.11 |
|  | H113 | *Scleromitrion diffusum* | Henan | cultivated | 20220901 | 0.1 | 2022.10.11 |
|  | H115 | *Scleromitrion diffusum* | Guangxi | wild | NO | 0.44 | 2022.10.11 |
|  | H117 | *Scleromitrion diffusum* | Guangxi | wild | NO | 0.5 | 2022.10.11 |
|  | H118 | *Scleromitrion diffusum* | Henan | wild | 20230214001 | 1.0 | 2323.03.10 |
|  | H119 | *Scleromitrion diffusum* | Henan | wild | 20230214002 | 1.0 | 2323.03.10 |
|  | H120 | *Scleromitrion diffusum* | Henan | wild | 20230214003 | 1.0 | 2323.03.10 |
|  | H121 | *Scleromitrion diffusum* | Hubei | wild | 20230214004 | 1.0 | 2323.03.10 |
|  | H122 | *Scleromitrion diffusum* | Hubei | wild | 20230214005 | 1.0 | 2323.03.10 |
|  | H123 | *Scleromitrion diffusum* | Henan | wild | 20230214006 | 1.0 | 2323.03.10 |
|  | H124 | *Scleromitrion diffusum* | Henan | wild | 20230214007 | 1.0 | 2323.03.10 |
|  | H125 | *Scleromitrion diffusum* | Hunan | wild | 20230214008 | 1.0 | 2323.03.10 |
|  | H126 | *Scleromitrion diffusum* | Hunan | wild | 20230214009 | 1.0 | 2323.03.10 |
|  | H127 | *Scleromitrion diffusum* | Henan | wild | 20230214010 | 1.0 | 2323.03.10 |
|  | H128 | *Scleromitrion diffusum* | Henan | wild | 20230214011 | 1.0 | 2323.03.10 |
|  | H129 | *Scleromitrion diffusum* | Henan | wild | 20230214012 | 1.0 | 2323.03.10 |
|  | H130 | *Scleromitrion diffusum* | Jiangxi | wild | 20230214013 | 1.0 | 2323.03.10 |
|  | H131 | *Scleromitrion diffusum* | Jiangxi | wild | 20230214014 | 1.0 | 2323.03.10 |
|  | H132 | *Scleromitrion diffusum* | Jiangxi | wild | 20230214015 | 1.0 | 2323.03.10 |
|  | NO | Marker***** | NO | NO | NO |  |  |
|  | NO | Asperuloside | NO | NO | NO |  |  |
|  | NO | Reference mixture | NO | NO | NO |  |  |
|  | NO | Standard herb | NO | NO | NO |  |  |
|  | H201 | *Scleromitrion diffusum* | Jiangxi | wild | 2202018 | 0.5 | 2022.09.07 |
|  | H202 | *Scleromitrion diffusum* | Guangdong | wild | 22070103 | 0.5 | 2022.09.07 |
|  | H203 | *Scleromitrion diffusum* | Fujian | wild | HX22F01 | 0.5 | 2022.09.07 |
|  | H204 | *Scleromitrion diffusum* | Guangdong | wild | / | 0.5 | 2022.09.07 |
|  | H205 | *Scleromitrion diffusum* | Henan | wild | C22207057 | 0.5 | 2022.09.07 |
|  | H206 | *Scleromitrion diffusum* | Henan | wild | C22112184 | 0.5 | 2022.09.07 |
|  | H207 | *Scleromitrion diffusum* | Henan | wild | C22207053 | 0.5 | 2022.09.07 |
|  | H208 | *Scleromitrion diffusum* | Henan | wild | C22207048 | 0.5 | 2022.09.07 |
|  | H209 | *Scleromitrion diffusum* | Hunan | wild | B2022413 | 0.5 | 2022.09.07 |
|  | H210 | *Scleromitrion diffusum* | Henan | wild | B2022618 | 0.5 | 2022.09.07 |
|  | H211 | *Scleromitrion diffusum* | Jiangxi | wild | 2207022 | 0.5 | 2022.10.11 |
|  | H212 | *Scleromitrion diffusum* | Hunan | wild | 220401 | 0.5 | 2022.10.11 |
|  | H213 | *Scleromitrion diffusum* | Hunan | wild | 220402 | 0.5 | 2022.10.11 |
|  | H214 | *Scleromitrion diffusum* | Hunan | wild | 220601 | 0.5 | 2022.10.11 |
|  | H215 | *Scleromitrion diffusum* | Hunan | wild | 220701 | 0.5 | 2022.10.11 |
|  | H216 | *Scleromitrion diffusum* | Hunan | wild | 220702 | 0.5 | 2022.10.11 |
|  | HC101 | *Hedyotis corymbosa* | Guangdong | wild | 2202018 | 0.02 | 2022.11.20 |
|  | HC102 | *Hedyotis corymbosa* | Hongkong | cultivated | 22070103 | 0.2 | 2023.02.06 |
|  | HC103 | *Hedyotis corymbosa* | Hongkong | wild | NO | 0.02 | 2023.09.13 |
|  | HC104 | *Hedyotis corymbosa* | Hongkong | wild | NO | 0.02 | 2023.09.13 |
|  | HC105 | *Hedyotis corymbosa* | Hongkong | wild | NO | 0.02 | 2023.09.19 |
|  | HC106 | *Hedyotis corymbosa* | Hongkong | wild | NO | 0.02 | 2023.06.26 |
|  | H134 | *Scleromitrion diffusum* | Hongkong | wild | NO | 0.02 | 2023.09.04 |
|  | H135 | *Scleromitrion diffusum* | Hongkong | wild | NO | 0.02 | 2023.09.06 |
|  | H136 | *Scleromitrion diffusum* | Hongkong | wild | NO | 0.02 | 2023.09.17 |
|  | H137 | *Scleromitrion diffusum* | Hongkong | wild | NO | 0.02 | 2023.09.25 |
|  | H114 | *Hedyotis tenelliflora* | Yunnan | wild | NO | 0.5 | 2022.10.12 |
|  | H116 | *Hedyotis tenelliflora* | Yunnan | wild | NO | 0.5 | 2022.10.12 |

**Table S2**. The contents of quality marker in commercial medicinal materials.

| No. | Weight (g) | Content (%) | Mean Content（%，n=4） |
| --- | --- | --- | --- |
| H101 | 2.0018 | 0.48 | 0.48 |
|  |  | 0.48 |  |
|  | 2.0035 | 0.48 |  |
|  |  | 0.48 |  |
| H102 | 2.0030 | 0.50 | 0.50 |
|  |  | 0.50 |  |
|  | 2.0034 | 0.50 |  |
|  |  | 0.50 |  |
| H103 | 2.0058 | 0.56 | 0.56 |
|  |  | 0.56 |  |
|  | 2.0026 | 0.57 |  |
|  |  | 0.56 |  |
| H104 | 2.0060 | 0.14 | 0.14 |
|  |  | 0.14 |  |
|  | 2.0056 | 0.14 |  |
|  |  | 0.14 |  |
| H105 | 2.0050 | 0.47 | 0.46 |
|  |  | 0.47 |  |
|  | 2.0058 | 0.45 |  |
|  |  | 0.45 |  |
| H106 | 2.0062 | 0.29 | 0.29 |
|  |  | 0.29 |  |
|  | 2.0021 | 0.28 |  |
|  |  | 0.28 |  |
| H107 | 2.0053 | 0.46 | 0.46 |
|  |  | 0.46 |  |
|  | 2.0048 | 0.45 |  |
|  |  | 0.45 |  |
| H108 | 2.0060 | 0.14 | 0.14 |
|  |  | 0.14 |  |
|  | 2.0056 | 0.14 |  |
|  |  | 0.14 |  |
| H109 | 2.0016 | 0.32 | 0.32 |
|  |  | 0.32 |  |
|  | 2.0062 | 0.33 |  |
|  |  | 0.33 |  |
| H110 | 2.0030 | 0.47 | 0.49 |
|  |  | 0.47 |  |
|  | 2.0044 | 0.51 |  |
|  |  | 0.51 |  |
| H111 | 2.0032 | 0.22 | 0.22 |
|  |  | 0.22 |  |
|  | 2.0064 | 0.22 |  |
|  |  | 0.22 |  |
| H113 | 2.0021 | 0.26 | 0.26 |
|  |  | 0.26 |  |
|  | 2.0051 | 0.26 |  |
|  |  | 0.26 |  |
| H117 | 2.0055 | 0.16 | 0.16 |
|  |  | 0.16 |  |
|  | 2.0051 | 0.16 |  |
|  |  | 0.16 |  |
| H118 | 2.0063 | 0.21 | 0.21 |
|  |  | 0.21 |  |
|  | 2.0072 | 0.21 |  |
|  |  | 0.21 |  |
| H119 | 2.0014 | 0.21 | 0.20 |
|  |  | 0.21 |  |
|  | 2.0034 | 0.20 |  |
|  |  | 0.20 |  |
| H120 | 2.0077 | 0.13 | 0.13 |
|  |  | 0.13 |  |
|  | 2.0061 | 0.13 |  |
|  |  | 0.13 |  |
| H121 | 2.0030 | 0.23 | 0.23 |
|  |  | 0.23 |  |
|  | 2.0069 | 0.23 |  |
|  |  | 0.23 |  |
| H122 | 2.0030 | 0.17 | 0.17 |
|  |  | 0.17 |  |
|  | 2.0025 | 0.17 |  |
|  |  | 0.17 |  |
| H123 | 2.0030 | 0.45 | 0.45 |
|  |  | 0.45 |  |
|  | 2.0045 | 0.45 |  |
|  |  | 0.45 |  |
| H124 | 2.0065 | 0.24 | 0.24 |
|  |  | 0.24 |  |
|  | 2.0051 | 0.24 |  |
|  |  | 0.24 |  |
| H125 | 2.0053 | 0.10 | 0.10 |
|  |  | 0.10 |  |
|  | 2.0023 | 0.10 |  |
|  |  | 0.10 |  |
| H126 | 2.0053 | 0.28 | 0.28 |
|  |  | 0.28 |  |
|  | 2.0025 | 0.28 |  |
|  |  | 0.28 |  |
| H127 | 2.0041 | 0.23 | 0.23 |
|  |  | 0.23 |  |
|  | 2.0041 | 0.23 |  |
|  |  | 0.23 |  |
| H128 | 2.0044 | 0.25 | 0.25 |
|  |  | 0.25 |  |
|  | 2.0071 | 0.25 |  |
|  |  | 0.25 |  |
| H129 | 2.0038 | 0.21 | 0.21 |
|  |  | 0.21 |  |
|  | 2.0074 | 0.21 |  |
|  |  | 0.21 |  |
| H130 | 2.0071 | 0.39 | 0.39 |
|  |  | 0.39 |  |
|  | 2.0033 | 0.40 |  |
|  |  | 0.40 |  |
| H131 | 2.0055 | 0.43 | 0.43 |
|  |  | 0.43 |  |
|  | 2.0060 | 0.43 |  |
|  |  | 0.43 |  |
| H132 | 2.0026 | 0.38 | 0.38 |
|  |  | 0.38 |  |
|  | 2.0071 | 0.38 |  |
|  |  | 0.38 |  |
| mean ± SD |  |  | 0.31 ± 0.13% |

**Table** **S3**. The contents of quality marker in decoction pieces.

| No. | Weight (g) | Content (%) | Mean Content（%，n=4） |
| --- | --- | --- | --- |
| H201 | 2.0062 | 0.54 | 0.54 |
|  |  | 0.53 |  |
|  | 2.0067 | 0.54 |  |
|  |  | 0.53 |  |
| H202 | 2.0040 | 0.42 | 0.42 |
|  |  | 0.42 |  |
|  | 2.0043 | 0.42 |  |
|  |  | 0.42 |  |
| H203 | 2.0081 | 0.40 | 0.41 |
|  |  | 0.40 |  |
|  | 2.0027 | 0.42 |  |
|  |  | 0.42 |  |
| H204 | 2.0025 | 0.38 | 0.38 |
|  |  | 0.38 |  |
|  | 2.0086 | 0.38 |  |
|  |  | 0.38 |  |
| H205 | 2.0055 | 0.34 | 0.33 |
|  |  | 0.34 |  |
|  | 2.0056 | 0.28 |  |
|  |  | 0.34 |  |
| H206 | 2.0056 | 0.25 | 0.25 |
|  |  | 0.25 |  |
|  | 2.0051 | 0.25 |  |
|  |  | 0.25 |  |
| H207 | 2.0018 | 0.31 | 0.31 |
|  |  | 0.31 |  |
|  | 2.0065 | 0.31 |  |
|  |  | 0.30 |  |
| H208 | 2.0069 | 0.35 | 0.35 |
|  |  | 0.35 |  |
|  | 2.0036 | 0.35 |  |
|  |  | 0.35 |  |
| H209 | 2.0012 | 0.19 | 0.19 |
|  |  | 0.19 |  |
|  | 2.0053 | 0.19 |  |
|  |  | 0.19 |  |
| H210 | 2.0056 | 0.30 | 0.30 |
|  |  | 0.30 |  |
|  | 2.0054 | 0.30 |  |
|  |  | 0.30 |  |
| H211 | 2.0054 | 0.25 | 0.25 |
|  |  | 0.25 |  |
|  | 2.0082 | 0.24 |  |
|  |  | 0.24 |  |
| H212 | 2.0026 | 0.30 | 0.31 |
|  |  | 0.30 |  |
|  | 2.0044 | 0.31 |  |
|  |  | 0.31 |  |
| H213 | 2.0056 | 0.31 | 0.32 |
|  |  | 0.31 |  |
|  | 2.0041 | 0.32 |  |
|  |  | 0.32 |  |
| H214 | 2.0046 | 0.33 | 0.33 |
|  |  | 0.33 |  |
|  | 2.0079 | 0.34 |  |
|  |  | 0.34 |  |
| H215 | 2.0038 | 0.32 | 0.32 |
|  |  | 0.33 |  |
|  | 2.0017 | 0.31 |  |
|  |  | 0.31 |  |
| H216 | 2.0079 | 0.29 | 0.30 |
|  |  | 0.30 |  |
|  | 2.0016 | 0.30 |  |
|  |  | 0.30 |  |
| Mean ± SD | - | - | 0.33 ± 0.08% |
